# Predictors of body image dissatisfaction in kinesiology students

**DOI:** 10.3389/fpsyg.2023.1322553

**Published:** 2024-02-02

**Authors:** Vedran Jakobek, Mislav Kranjčev, Renata Barić

**Affiliations:** ^1^Faculty of Kinesiology, University of Zagreb, Zagreb, Croatia; ^2^Independent Researcher, Zagreb, Croatia

**Keywords:** body image dissatisfaction, sex differences, perfectionism, self-esteem, eating attitudes

## Abstract

**Introduction:**

This research aimed to examine predictors of discrepancies between actual and ideal body images, specifically body fat and muscularity discrepancies, in kinesiology students.

**Methods:**

Volunteer participants (*N* = 174, men = 112, women = 62) filled out the sex-specific Somatomorphic Matrices (NSM-M and SM-F) as well as The Positive and Negative Perfectionism Scale (PNPS), Rosenberg Self-esteem Scale (RSE), and the Eating Attitudes Test (EAT-26) using paper and pencil tools.

**Results:**

The median and Mann–Whitney *U* tests were used to determine sex differences, and regression analyses were used to determine the contribution of sex, perfectionism, self-esteem, and eating attitudes in explaining the variances in body fat and muscularity dissatisfaction. The results showed no sex differences in body fat discrepancy, while men showed more discrepancy between actual and ideal muscularity than women. In regression analyses, sex was the only significant predictor of muscularity discrepancy, while disordered eating attitudes were a positive, and positive perfectionism was a negative predictor of body fat discrepancy.

**Discussion:**

This study showed that while muscularity dissatisfaction is linked to the male sex, body fat dissatisfaction is not related to sex but to psychological variables of disordered eating attitudes and positive perfectionism in a population of youths that may be above average when it comes to physical activity.

## Introduction

Body image is one of many aspects of an individual’s idea of self, and dissatisfaction with one’s body image can be especially salient in adolescents and young adults (O’Dea, 2012). Athletes and young people involved in sports, such as kinesiology students, are at special risk for body image disturbance, due to task and social pressures to achieve an ideal physique (Hausenblas and Downs, 2001). It could be particularly prominent in the age of technological growth and the wide spread of social networking sites’ usage, as comparisons with others, including comparisons of physical appearance, seem to grow more frequent. Kirkpatrick and Lee (2023) suggest that participating in social networking sites, especially image-based ones, could have negative effects on body image satisfaction in youth, with Carmona et al. (2015) and Cox et al. (2017) noting that media has a larger influence on youths’ body image than other social influences such as family or friends. It is, therefore, of scientific interest to thoroughly explore body image satisfaction as the evaluative aspect of one’s body and its links to relevant areas in sports psychology.

Body image is a multidimensional attitude toward one’s body, especially its appearance (Cash and Pruzinsky, 1990; Muth and Cash, 1997). Cash (1994, 2004) discusses that three aspects of attitudes toward one’s body should be distinguished: evaluative, affective, and investment. The evaluative aspect of body image refers to (dis)satisfaction with physical attributes and evaluative thoughts and beliefs about one’s appearance. These evaluations partly derive from discrepancies of self-perceived current appearance from internalized body ideals (see Muth and Cash, 1997). The affective aspect refers to emotions related to appearance, while the investment aspect refers to the importance a person places on appearance (Cash, 1994). Body image research is important because dissatisfaction with one’s body can result in unhealthy behaviors, including maladaptive eating and exercise patterns (Anton et al., 2000; Grogan, 2006).

Body image dissatisfaction can be defined as a negative subjective evaluation of one’s own body as a whole or concerning specific aspects of the body, such as height, shape, muscularity, or weight (Grogan, 2016). Empirical results suggest that women express higher body image dissatisfaction than men (Muth and Cash, 1997; Demarest and Allen, 2000; Leng et al., 2020). However, it is incorrect to assume that body dissatisfaction exists only in women. Research indicates that body image dissatisfaction in the male population has been on the rise in recent years (see Talbot et al., 2020). Although there are some similarities, body image dissatisfaction manifests differently in men and women. This difference can be related to the shape and composition of what men and women consider the ideal body. Most men think a mesomorphic body type to be ideal (Talbot et al., 2020). Such a body type is defined by a low percentage of body fat combined with defined, visible muscles (developed muscles of the chest, shoulders, and arms; narrow waist and hips, and a V-shaped torso; see Talbot et al., 2020). In the Western world, most women idealize a low body fat percentage a narrow waist, and strive for an hourglass figure. Women’s ideal muscularity differs from men’s in that they often strive for defined, but smaller muscles (see Talbot et al., 2020). Considering body image dissatisfaction in the context of body fat and muscularity levels, both sexes may be dissatisfied with body fat percentage, waist size, and muscle tone (Talbot et al., 2020). Furthermore, men’s dissatisfaction will stem more from the shape of muscles and their size, while body dissatisfaction in women will be more characterized by how slim their body is (Grogan, 2016). Valls et al. (2013) found that about 85% of male French youths included in the study were dissatisfied with their levels of muscularity. This article aims to cover multiple aspects of body image dissatisfaction, including potential biological, psychological, and behavioral predictors. Special interest was put in potential psychological correlates that are being extensively examined in other areas of sports psychology as important for both individual well-being and sports performance, such as self-esteem (Ouyang et al., 2020) and perfectionism (Stoeber, 2011), as well as disordered eating attitudes (Kong and Harris, 2015) which are documented to be highly related to body image and relevant for both athlete and non-athlete populations.

Perfectionism can be described as a person’s effort to be flawless, whereby very high functioning parameters are established in a certain context, with a tendency to self-criticism when evaluating behavior (Frost et al., 1990; Flett and Hewitt, 2002). Some authors divide perfectionism into positive and negative^1^ (Terry-Short et al., 1995). The positive dimension of perfectionism refers to facets associated with perfectionistic aspirations, such as setting high personal standards and focusing on excellence. The negative dimension of perfectionism refers to facets associated with perfectionistic concerns, such as worries about making a mistake, doubting one’s performance, feeling a discrepancy between expectations and results, and negative reactions to mistakes (Stoeber et al., 2007). In the sports context, positive perfectionism was associated with a higher level of training performance (Madigan et al., 2018a), as well as with a lower level of stress experienced during training (Madigan et al., 2018b), while negative perfectionism seems to be a risk factor for the development of exercise dependency (Costa et al., 2016). There are several studies in which facets of perfectionism have been associated with body image dissatisfaction (Grammas and Schwartz, 2009; Sherry et al., 2009). In a study conducted on a sample of kinesiology students, positive perfectionism was positively correlated with body satisfaction in a subsample of women, and negative perfectionism was negatively correlated with body satisfaction in both subsamples of women and men (Prnjak et al., 2019). Perfectionism is a personality trait defined by the pursuit of high standards and critical assessment of oneself in various areas, including body image satisfaction, which is closely related to mental health and profoundly impacts an individual’s life (Fang and Liu, 2022). Failure to achieve the desired body image in young people may be associated with maladaptive psychological states such as anxiety (Vannucci and Ohannessian, 2018), depressive symptoms (Soares Filho et al., 2020), or low self-esteem (Van Den Berg et al., 2010).

Global self-esteem is a comprehensive positive or negative attitude about oneself (Rosenberg et al., 1995). Self-concept theories assume that dissatisfaction in a particular domain will impact global self-esteem to the extent that domain is central to a person’s definition of self (see Tiggemann, 2005). According to the theory of contingencies of self-worth, proposed by Crocker et al. (2003), there are seven specific sources of self-esteem in students, and physical appearance is one of them. Some authors argue that there are sex differences in the internalized degree of importance given to appearance and body weight, which is higher in women (Rodin et al., 1984). The direction of causality in the relationship between body dissatisfaction and low self-esteem has not yet been fully clarified (Tiggemann, 2005). According to theories that conceptualize global self-esteem as a composite that includes different domains important to the individual, body image dissatisfaction is an antecedent of (low) self-esteem. On the other hand, etiological theories of negative body image development propose a model in which low self-esteem precedes body image dissatisfaction, either directly or indirectly (e.g., through an unrealistic idea of how the body should look; Tiggemann, 2005). Self-esteem is a factor that is particularly important when considering reactions to failure in achieving desired goals (such as an ideal body image) and can be lowered if goals are not reached (Zogmaister and Maricutoiu, 2022). To get closer to the desired body image, young people may engage in weight control behaviors such as restrictive eating (Ben Ayed et al., 2021). However, it is suggested that failure to identify behaviors that are overly restrictive or controlling can lead to eating disorder symptoms (Juarascio et al., 2020). The interaction between perfectionism and eating disorders is particularly pronounced, whereby people with higher perfectionist standards try to control eating habits, body shape, and weight to a greater extent (Fairburn et al., 2003).

Research consistently indicates a positive correlation between body image dissatisfaction and disordered eating behaviors and attitudes (Mayo and George, 2014; Yang et al., 2022). Disordered eating symptomatology includes behaviors and attitudes^2^ such as strict diets, overeating, preoccupation with food, etc. Such habits can impair life quality (Welch et al., 2009). Although playing sports is associated with various physiological and psychosocial benefits, research suggests that, compared to non-athletes, the prevalence of disordered eating symptomatology is higher in the population of athletes (see Kong and Harris, 2015). Some authors (e.g., Kong and Harris, 2015) assume that body image dissatisfaction and disordered eating attitudes in athletes may depend on the type of sport and the level at which the athlete competes. The assumptions are that athletes who compete in aesthetic sports will report a higher level of body image dissatisfaction and more pronounced disordered eating attitudes than athletes who compete in non-aesthetic sports. Further, elite athletes will report greater body image dissatisfaction and more pronounced disordered eating symptomatology compared to recreational athletes (Kong and Harris, 2015).

This research aims to examine sex differences in dissatisfaction with body fat and muscularity levels in a sample of kinesiology students. Furthermore, potential biological, psychological, and behavioral predictors of body image dissatisfaction will be discussed in the context of these two aspects of physique. We formulated several hypotheses based on the results of previously mentioned research. We expected to find sex differences in body image dissatisfaction, with women showing greater body fat dissatisfaction and men showing greater muscularity dissatisfaction. We expected female sex, negative perfectionism, and disordered eating attitudes to act as positive predictors of body fat dissatisfaction. We expected positive perfectionism and self-esteem to negatively predict body fat dissatisfaction. We expected male sex, negative perfectionism, and disordered eating attitudes to act as positive predictors of muscularity dissatisfaction. We expected positive perfectionism and self-esteem to be negative predictors of muscularity dissatisfaction.

## Method

### Sample

Students (*N* = 174) who attended the Elements of Psychology course in the 2nd year of the Faculty of Kinesiology at the University of Zagreb participated in the research. The majority (*N* = 112) of the participants were males. The participants’ average age was 20.30 years (*SD* = 0.80). A third of the participants (33.33%) stated that they did not play sports outside of their university sports, while the others played football (16.09%), basketball (7.47%), handball (6.90%), or attended a gym (6.90%). The average weekly time spent practicing or competing in sports outside of faculty duties was 9.56 h (*SD* = 4.12). The majority of participants (51.72%) do not participate in sports competitions. In comparison, the rest of them compete at the regional level (20.69%), national level (15.52%), international level for a club (4.02%), or are members of the national team^3^ (6.90%). More of the participants’s characteristics are presented in Table 1.

**Table 1:** Descriptive data of the variables used in the research (*N* = 174).

	*N*	*M*	*SD*	*Min*	*Max*	*Skew*	*Kurt*	*S-W*	*p_S-W_*
Age	174	20.30	0.80	19.00	24.00	1.42	3.46	-	-
Weight (kg)	174	74.69	12.73	48.00	112.00	0.13	−0.57	-	-
Height (cm)	174	178.51	9.16	156.00	203.00	−0.10	−0.40	-	-
BMI	174	23.28	2.37	17.99	29.00	0.05	−0.59	-	-
Body fat dissatisfaction	169	9.00	14.33	−18.37	54.42	0.89	0.65	0.93	< 0.01
Muscularity dissatisfaction	169	19.24	15.15	−35.74	62.98	0.21	0.62	0.97	< 0.01
Positive perfectionism	170	76.19	7.80	59.00	95.00	0.24	−0.38	0.99	0.14
Negative perfectionism	171	52.94	10.39	30.00	79.00	0.07	−0.60	0.99	0.30
Self-esteem	171	30.60	4.74	17.00	40.00	−0.30	−0.28	0.98	0.04
Disordered eating attitudes	169	55.53	13.20	26.00	101.92	0.61	0.63	0.91	< 0.01

*N*, number of participants with valid data for a particular variable; *M*, mean; *SD*, standard deviation; *Min*, minimum result; *Max*, maximum result; *Skew*, skewness; *Kurt*, kurtosis; *S-W*, Shapiro–Wilk test value; *p*_*S-W*_, significance level of the Shapiro–Wilk test; kg, kilograms; cm, centimeters; BMI, body mass index (calculated via formula: body mass in kilograms divided by the square of the height in meters) based on self-reported weight and height.

With statistical procedures being selected based on preliminary analyses, the Mann–Whitney *U* tests showed that there were no differences in body fat dissatisfaction between those who play sports outside faculty duties vs. those who do not (*U* = −0.25; *p* > 0.05), as well as between those who compete vs. those who do not compete in sports outside faculty duties (*U* = −0.30; *p* > 0.05). The median tests showed that there were no differences in muscularity dissatisfaction between those who play sports outside faculty duties vs. those who do not (*Z* = −0.11; *p* > 0.05), as well as between those who compete vs. those who do not compete in sports outside faculty duties (*Z* = −0.22; *p* > 0.05). This justifies the inclusion of participants from these subgroups in the joint analyses and the research findings are likely to generalize to these subgroups.

### Instruments

Firstly, the participants filled out a short sociodemographic questionnaire, which included questions about age, biological sex, height, and weight. Participants were also asked to indicate the sport they play, the length of time (in years) they have played the sport, the level at which they compete, and the average time per week (in hours) they spend playing that sport.

Dissatisfaction with body appearance was measured using (New) Somatomorphic Matrices. The male (NSM-M; The New Somatomorphic Matrix-Male; Talbot et al., 2019) and female (SM-F; The Somatomorphic Matrix-Female; Talbot et al., 2023) versions of the matrices were used in the research. These questionnaires represent two-dimensional scales on which 34 images of male or female bodies (which vary in body fat and muscularity) are shown. Participants were asked to mark any location on the grid that best corresponds to their (a) actual body and (b) ideal body. Body representations on the scales varied as a function of body fat on the x-axis and muscularity on the y-axis. The length of the x-axis was 14.7 cm, and the length of the y-axis was 23.5 cm. The results on the two dimensions were transformed to a scale ranging from 0 to 100 (e.g., a point 14.7 cm from the origin on the x-axis got a score of 100, and 13 cm from the origin on the y-axis got a score of 13 / 23.5 * 100 = 55.32). The discrepancy for the body fat dimension was calculated by subtracting the desired results from the actual results. The discrepancy for muscularity was calculated by subtracting the actual results from the desired results. The discrepancy between actual and ideal body appearance is conceptualized as an index of body dissatisfaction, spread across the aspects of body fat and muscularity, with a larger discrepancy indicating a higher level of body dissatisfaction (Gardner and Brown, 2010). However, one should be aware that this kind of conceptualization may be questionable. Most people probably think their ideal body would be somewhat different (e.g., leaner and/or more muscular) than their actual body. However, someone can want a particular body type without necessarily being dissatisfied with their own body (Talbot et al., 2020). With this in mind, discrepancy in figural measures of body satisfaction correlates highly with various measures related to body image dissatisfaction, including disordered eating symptoms (Smith et al., 2011), drive for muscularity (Novella et al., 2015), and body appreciation (Mutale et al., 2016). According to Talbot et al. (2020), for this reason, figure scales, such as NSM-M and SM-F, which enable the calculation of the discrepancy between the perception of the actual and ideal body appearance, are a valuable tool for measuring body dissatisfaction.

Self-esteem was measured with the Croatian version (Lacković-Grgin, 1994) of the Rosenberg self-esteem scale (RSE; Rosenberg, 1965). The scale consists of 10 items (e.g., “I feel that I have a number of good qualities.”). On a 4-point Likert-type scale (1 – strongly disagree; 4 – strongly agree), the participants reported how much a particular statement applied to them (with five items being reverse scored). A higher total score indicates higher self-esteem. A satisfactory level of reliability of the scale was determined by Cronbach’s alpha coefficient (*α* = 0.84), which is in accordance with the results of earlier research (e.g., Kuterovac, 2022).

Perfectionism was measured with the Croatian version (Lauri Korajlija, 2004) of the Positive and Negative Perfectionism Scale (PNPS; Terry-Short et al., 1995). The scale consists of 40 items, half of which measure positive (e.g., “My successes spur me on to greater achievements.”) and half negative (e.g., “I feel guilty or ashamed if I do less than perfectly.”) perfectionism. On a 5-point Likert-type scale (1 – strongly disagree; 5 – strongly agree), the participants assessed the extent to which a particular statement applied to them. A higher score indicates higher perfectionism. In this research, a satisfactory level of reliability of the subscales was established. Cronbach’s alpha value was 0.78 for the subscale measuring positive perfectionism and 0.85 for the subscale measuring negative perfectionism. The satisfactory reliability of the subscales is in line with the results of previous research (e.g., Kapetanović, 2008).

The eating attitudes of the participants were measured with the Croatian version (Ambrosi-Randić and Pokrajac-Bulian, 2005) of the Eating Attitudes Test (EAT-26; Garner et al., 1982). Although the original version of the questionnaire allows the measurement of three dimensions, in this study, the total score was used as a measure of the presence of some symptoms of an eating disorder. The questionnaire consists of 26 items (e.g., “I find myself preoccupied with food.”). On a 6-point Likert-type scale (1 – never, 6 – always), the participants assessed the extent to which a particular statement applied to them (the 26th particle is scored inversely). EAT-26 is a triage instrument whose most common purpose is to examine behaviors and attitudes that occur in anorexia and bulimia nervosa (Pokrajac-Bulian et al., 2004). When used for triage purposes, Garner and Garfinkel (1979) suggest that the instrument is scored in a way that answers from 1 to 3 are assigned 0 points, and answers from 4 to 6 are assigned 1–3 points. When the instrument is scored in this way, a critical score of 20 or more points could be clinically significant (Garner et al., 1982). However, in this study we decided to keep the full range of scores, so we assigned scores 1–6 to answers 1–6. Keeping the full range better reflects the variation among participants’ answers, but it is important to note that such overall results (and, for example, their mean and standard deviation) are not directly comparable to those obtained with the original scoring (however, the results calculated by the two scoring methods are highly correlated, with Pearson’s *r* of 0.82). A higher score on the questionnaire indicates a greater expression of disordered eating attitudes (Pokrajac-Bulian et al., 2005). Reliability, when the results are scored in the full range, in this sample was 0.57, which is below the limit that is traditionally considered acceptable (Nunnally, 1978). It is possible that in this sample, disordered eating attitudes are not unidimensional as they are in the general population. Nevertheless, in some earlier studies (e.g., Pokrajac-Bulian et al., 2004) in which the Croatian version of the scale was used, satisfactory reliability was determined.

### Procedure

The data were collected during January of 2023. in the Elements of Psychology classes at the Faculty of Kinesiology, University of Zagreb. The research was conducted via paper and pencil. Participation in the study was voluntary and anonymous, and participants could withdraw from participation at any time during the research process. The average duration of filling out the questionnaire was about 10 minutes. For their effort, students were awarded points that are considered when forming the final grade for the course Elements of Psychology. Questionnaires were filled out during the Elements of Psychology classes, and rewards for participation were assigned by a teaching assistant who was not a part of the research team based on class attendance and not by checking for questionnaire completion, therefore preserving anonymity. The matrices were scored on two occasions by two researchers, and eventual scoring deviations were discussed and corrected. The rest of the questionnaires were scored by hand and controlled by entering individual items onto the computer and calculating total scores digitally so that mismatches between hand-calculated and digitally-calculated total scores would point to calculation errors which would then be corrected. Still, no such errors occurred.

The statistical plan included a data quality check. After the preliminary data analysis, it was established that 5 participants did not fill out the materials correctly or did not fill them out completely. If the participant did not answer more than one question per subscale of the questionnaire, the result of the subscale of that participant was excluded from the processing. For 165 out of 174 participants, data is available for all investigated measures. The observed measures were to be checked for normality, and the analysis plan was to compute the descriptive statistics, correlation coefficients (Pearson or Spearman, depending on normality test results), and two regression analyses with the same sets of predictors, with one having body fat dissatisfaction and the other muscularity dissatisfaction as the criterium. The method of calculating sex differences was to be selected depending on the normality of distributions and equality of variances in the body fat and muscularity dissatisfaction in subsamples of men and women. Data processing was performed in the statistical programming language R (R Core Team, 2022).

## Results

The descriptive statistics for the variables used in this research are displayed in Table 1. Before comparing levels of body fat dissatisfaction between sexes, the normality of the distributions of the results was tested, as well as the equality of variances for the subgroups of men and women (Table 2). The results of the Shapiro–Wilk test indicate the non-normality of the distribution of results in the variable body fat dissatisfaction in the subgroup of men. Analysis of variance determined that the variances in the two groups were not equal (*F* = 2.52; *p* < 0.01). Based on the previously performed analyses, a decision was made to perform a median test to examine sex differences in body fat dissatisfaction. The results of the median test (*Z* = −0.06; *p* > 0.05) show that there is no difference in body fat dissatisfaction between men and women.

**Table 2:** Presentation of descriptive statistics for body fat and muscularity dissatisfaction for subgroups of men and women (*N* = 169).

		*M*	*SD*	*Min*	*Max*	*S-W*	*p_S-W_*
Body fat dissatisfaction	Men (*N = 107*)	9.85	16.23	−18.37	54.42	0.92	< 0.01
	Women (*N = 62*)	7.55	10.22	−15.65	36.73	0.98	0.34
Muscularity dissatisfaction	Men (*N = 107*)	22.05	13.61	−0.43	55.74	0.95	< 0.01
	Women (*N = 62*)	14.38	16.50	−35.74	62.98	0.97	0.19

*N*, number of participants; *M*, mean; *SD*, standard deviation; *Min*, minimum result; *Max*, maximum result; *S-W*, Shapiro–Wilk test value; *p_S-W_*, significance level of the Shapiro–Wilk test.

Before comparing levels of muscularity dissatisfaction between the sexes, the normality of the distributions of the results was tested, as well as the equality of variances in the subgroups of men and women. The results of the Shapiro–Wilk test indicate the non-normality of the distribution of results for the subgroup of men in the variable muscularity dissatisfaction (Table 2). Analysis of variance revealed that the variances in the two groups are equal (*F* = 0.68; *p* > 0.05). Based on previously conducted analyses, the difference between the groups was tested using the Mann–Whitney *U* test. The results of the Mann–Whitney *U* test (*U* = 3.21, *p* < 0.01) indicate a statistically significant difference in muscularity dissatisfaction between the sexes. Men (*M* = 22.05) reported higher dissatisfaction levels than women (*M* = 14.38). The difference between the sexes is statistically significant even when the correction for multiple comparisons was applied (e.g., Bonferroni’s correction).

Table 3 contains bivariate Spearman’s correlation coefficients calculated on the variables used in this study. Body fat dissatisfaction is positively associated with disordered eating attitudes. Muscularity dissatisfaction is positively associated with the male sex. The association between body fat dissatisfaction and muscularity dissatisfaction did not reach statistical significance.

**Table 3:** Presentation of Spearman’s correlation coefficients between the variables (*N* = 174).

	1.	2.	3.	4.	5.	6.	7.
1. Sex^1^	-	−0.01	−0.25**	0.01	0.04	−0.09	0.10
2. Body fat dissatisfaction		-	0.11	−0.14	0.13	−0.14	0.20*
3. Muscularity dissatisfaction			-	−0.09	0.03	−0.09	0.04
4. Positive perfectionism				-	0.25**	0.18*	0.10
5. Negative perfectionism					-	−0.61**	0.39**
6. Self-esteem						-	−0.31**
7. Disordered eating attitudes							-

**p < 0.01; * *p* < 0.05.

^1^Sex was coded so that men were associated with the number 1 and women with the number 2.

In order to determine the contribution of sex, perfectionism, self-esteem, and eating attitudes in explaining the variance of body fat dissatisfaction, a hierarchical regression analysis was performed.^4^ The variables were included in the analysis in two blocks. The first block included sex, while the second block also included perfectionism, self-esteem, and disordered eating attitudes. Before conducting the hierarchical regression analysis, the tolerance and variance inflation factor (VIF) values of the predictors were inspected to determine potential multicollinearity. All VIF values were between 1.0 and 2.1 and the lowest tolerance was 0.47. VIF values above 10 and tolerance values below 0.1 are considered problematic and indicate multicollinearity (Miles, 2014). Therefore, no significant collinearity was found among the potential predictors.

The overall *R*^2^ suggests that positive perfectionism and disordered eating attitudes can explain 10% of the variance in body fat dissatisfaction. The first block, in which sex is included, does not contribute significantly to explaining the variance of the criteria. After the inclusion of the second block of variables, positive perfectionism proved to be a negative predictor, and disordered eating attitudes a positive predictor of body fat dissatisfaction (Table 4).

**Table 4:** Results of hierarchical regression analyses for the criteria body fat dissatisfaction and muscularity dissatisfaction (*N* = 169).

	Criterion: body fat dissatisfaction		Criterion: muscularity dissatisfaction	
	*β*M1	*β*M2	*β*M1	*β*M2
Sex	−0.08	−0.10	−0.25**	−0.27**
Positive perfectionism		−0.27**		−0.10
Negative perfectionism		0.12		−0.03
Self-esteem		0.08		−0.04
Disordered eating attitudes		0.20*		0.13
*R* ^2^	0.01	0.10	0.06	0.09
*F*	1.04	3.46	10.97	3.26
*P*	0.31	< 0.01	< 0.01	< 0.01
Δ*R*^2^	-	0.09	-	0.03
*F*Δ*R*^2^	-	4.05	-	1.31
*p*Δ*R*^2^	-	< 0.01	-	0.27

*β*, the value of the standardized regression coefficient; M1, M2, groups of predictors in hierarchical regression analysis (models); *R*^2^, the total contribution to the explained variance; *F*, the F-ratio value; *p*, significance level of the model; Δ*R*^2^, the contribution of the additional group of predictors to the explained variance; *FΔR*^2^, the F-ratio value for the additional group of predictors; *p ΔR*^2^, the significance level of the increase in the explained criterion variance with the inclusion of an additional group of predictors; **p < 0.01; * *p* < 0.05.

To determine the contribution of sex, perfectionism, self-esteem, and eating attitudes in explaining the variance of muscularity dissatisfaction, a hierarchical regression analysis was performed with the same predictor groupings as for body fat dissatisfaction. The overall *R*^2^ suggests that sex can explain 6% of the variance in body fat dissatisfaction. Male sex is associated with greater muscularity dissatisfaction. The inclusion of psychological and behavioral variables in the second block does not significantly contribute to explaining the variance of the criteria.

## Discussion

The aim of this research was to test sex differences in body image dissatisfaction, as well as its predictors, in a sample of students of the Faculty of Kinesiology in Zagreb. Body image dissatisfaction in this study included two aspects of physique: body fat and muscularity. In the present sample, the results show no differences between men and women regarding body fat dissatisfaction. In contrast, men report a higher level of muscularity dissatisfaction compared to women. Positive perfectionism and disordered eating attitudes were statistically significant predictors of body fat dissatisfaction, while sex was the only statistically significant predictor of muscularity dissatisfaction.

**Figure 1:**
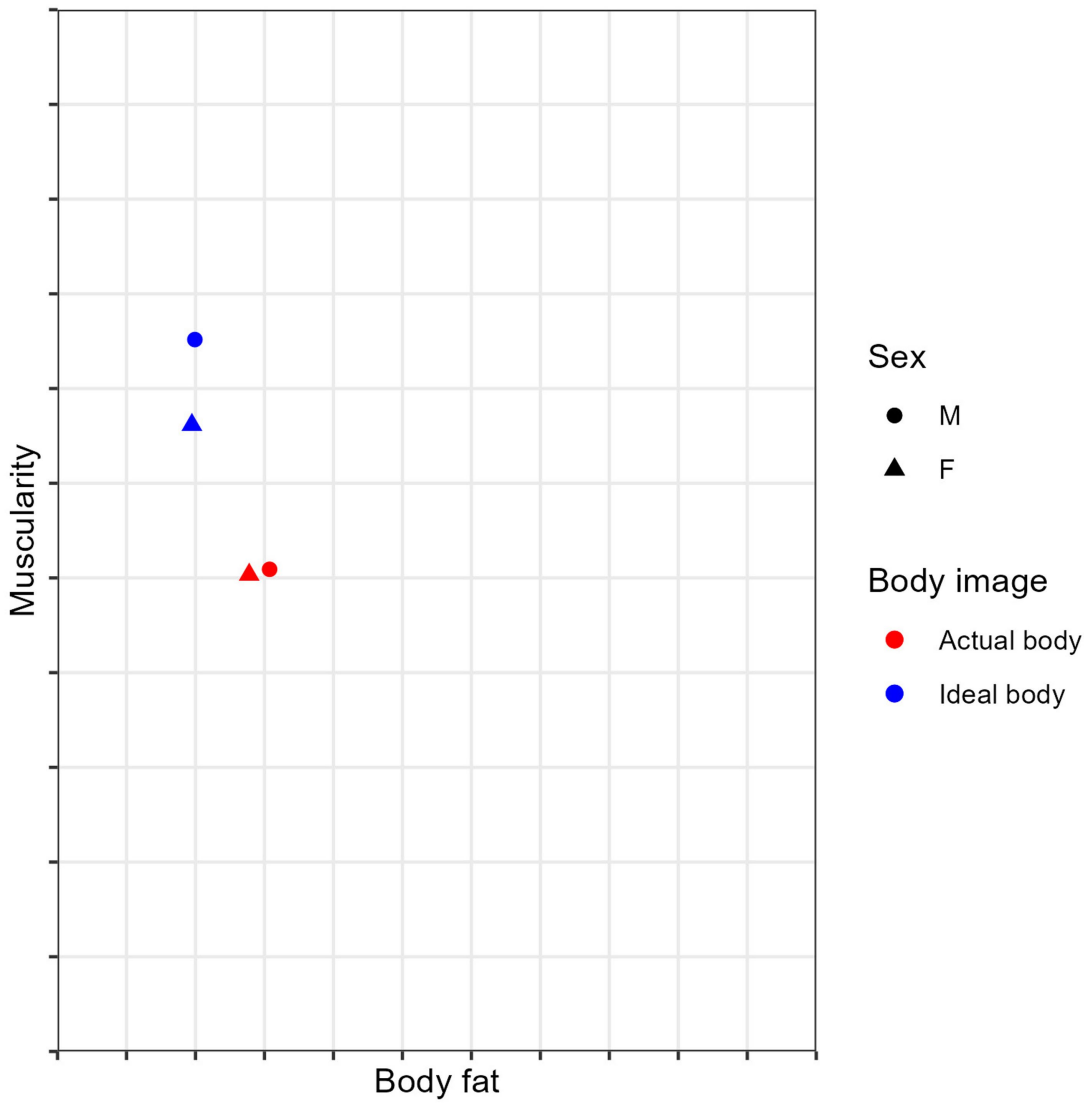
Approximate representation of the average response of participants for subsamples of women (*N* = 62) and men (*N* = 107) in the task of labeling their actual and ideal bodies. See Talbot et al. (2019) for the original version of the NSM-M, and Talbot et al. (2023) for the original version of the SM-F.

Talbot et al. (2019), in a paper validating the male version of the Somatomorphic Matrix, state that the models in the images within the grid span a range of 4 to 40% body fat in increments of 4%. The fat-free mass index (muscularity) ranges from 16.5 to 30 kg/m^2^ and increases in 1.5 kg/m2 increments. Data for the range of body fat and muscularity dimensions of the models in the female version of the scale is unavailable (Talbot et al., 2023). The present sample consists of physically active people, many of whom, in addition to their college duties, which include physical activity, also play sports. Research consistently shows that physical activity negatively correlates with body fat levels (Besson et al., 2009; Bradbury et al., 2017). Based on the data available for the male version of the scale (Talbot et al., 2019), we can speculate that the average self-estimated body fat percentage of men in our sample is around 12% (see Figure 1). Due to the relatively low self-estimates of actual body fat (see reference values in Kesavachandran et al., 2009), the ideal level of body fat (which in this study is in almost all cases lower than actual body fat) did not differ much from the actual body fat. Similar results were found in the female subsample. We believe that this low self-estimated level of actual body fat in both subsamples is why no sex differences were found in body fat dissatisfaction. For example, we would expect more cases of a large discrepancy between actual and ideal fat levels in the general population. Our results show differences between men and women in muscularity dissatisfaction. According to research so far (Grogan, 2016), body image dissatisfaction will be determined by muscle shape and size to a larger degree for men than for women, and our findings support this notion.

The results of the hierarchical regression analysis show that a higher level of positive perfectionism is associated with a lower level of body fat dissatisfaction. Parallels can be made between this finding and those of Prnjak et al. (2019) on a sample of students of the Faculty of Kinesiology. In their research, positive perfectionism was positively correlated with body image satisfaction in female students. In the sports context, research has shown that positive perfectionism is positively related to several different adaptive outcomes (Madigan et al., 2018a,b). People with such a perfectionist orientation set achievable goals and strive to fulfill them (Enns et al., 2002). Regarding body fat dissatisfaction, it is possible that individuals characterized by a higher level of positive perfectionism will have more realistic demands related to body fat percentage and will be persistent in achieving these demands. Gao et al. (2023) analyzed similar psychological phenomena, e.g., perfectionism and self-esteem, in dental medicine. They studied a narrower scope of body image, focusing on one’s orofacial appearance and concern about the appearance of one’s smile. Their results point to higher perfectionism being linked to more worry about one’s appearance, higher subjective importance of body image, and worse self-esteem. These findings initially do not seem in line with ours and those reported by Prnjak et al. (2019). However, in the research by Gao et al. (2023), perfectionism was measured by a different instrument that conceptualizes perfectionism closer to what has been called “negative perfectionism” in this study. Therefore, positive perfectionism might be related to higher body image satisfaction, probably owing to adaptive behaviors fostered by positive perfectionism, while negative perfectionism in some samples could be related to higher levels of body image dissatisfaction and worse self-esteem. A correlational study by Teixeira et al. (2016) on a large sample of female high-school students in Portugal also linked higher levels of (negative) perfectionism to lower self-esteem and higher body image dissatisfaction, as well as more disordered eating behaviors.

In this study, disordered eating attitudes were shown to predict body fat dissatisfaction. These variables have been shown to be related in various studies in the context of sports (e.g., de Souza Fortes et al., 2015). Anton et al. (2000) have found that the size of the discrepancy between actual and ideal appearance is related to disordered eating attitudes in female students. It should be emphasized that, given the nature of the research, we cannot make conclusions about the causal influence of disordered eating attitudes on body fat dissatisfaction. Moreover, researchers dealing with this issue often theorize that body image dissatisfaction is a risk factor for the development of disordered eating attitudes (Tylka, 2004; de Souza Fortes et al., 2013; Wade and Tiggemann, 2013). According to the theoretical model of disordered eating habits in athletes (Petrie and Greenleaf, 2007), sociocultural factors, combined with sports-related pressure, can lead to body image dissatisfaction, which then leads to disordered eating.

Research results indicate that body appearance pressure is a pronounced problem among the student population (Sundgot-Borgen et al., 2021). Real and apparent peer pressure for a lower body fat percentage and higher muscularity may be more pronounced among kinesiology faculty students than among the general population. Tylka (2011) empirically tested model suggests that perceiving pressure from friends for mesomorphic body shape is directly related to increased muscularity dissatisfaction in men. It has also been shown that muscularity dissatisfaction mediates the association between perceived friend pressure and muscularity enhancement behaviors in men (Tylka, 2011). We hypothesize that in the population of already physically active people, higher body image dissatisfaction, especially accompanied by higher perfectionism and lower self-esteem levels, could lead to overly excessive muscularity enhancement behaviors, which could result in undesired outcomes like overtraining. It is well-documented that overtraining is accompanied by maladaptive psychological and immunological deficits (Cadegiani, 2020). What has also been shown is that family and media pressures for a mesomorphic appearance predict the internalization of the mesomorphic ideal. Internalization of the mesomorphic ideal then mediates the relationships between family and media pressure for the mesomorphic ideal and muscularity and body fat dissatisfactions in men (Tylka, 2011). It is possible that, due to the sports nature of their academic orientation, kinesiology students spend more time searching for fitness-related social media or other online content than the general population. In this realm, popular content includes fitness influencers whose bodies are close to what people could consider ideal. Because of that, they may internalize mesomorphic body ideals to a great extent. Research (see Kearney, 2023) confirms that exposure to idealized photos on social networking sites may make people more dissatisfied with their bodies, which may make kinesiology students more at risk of developing body image dissatisfaction.

### Limitations and directions for future research

The sample in this research consisted of students of the Faculty of Kinesiology, who represent a specific group of physically active people, making the research findings relevant to sports psychology. However, these participants represent a population that differs from the general population in certain characteristics, which may affect the generalization of the findings. The range of body mass index in this sample is relatively narrow. This is probably why the differences between actual and ideal body fat levels are smaller (since the average actual body fat level self-reported in this sample is relatively low). Also, the age range of the participants is very narrow. The majority of participants are between the ages of 19 and 21, so it was impossible to determine the potential relationship between age and other variables. Future research that covers a broader age range would help determine differences in actual and ideal body image discrepancy related to age in more detail. Data on height and weight were collected based on the participants’ self-assessments, and BMI was then calculated based on this data. If height and weight were measured more accurately (i.e., by measuring participants’ height and weighting them), BMI calculated via these measures would be more valid and a variable of interest in future research.

Research suggests that some factors, such as sexual orientation (Meneguzzo et al., 2022) or internalization of social norms about ideal body shape (Stewart, 2021), may also be related to cognitive bias about one’s body image. To control such biases, these variables could be included in future research on body image dissatisfaction in kinesiology students. Recent research conducted by Leng et al. (2020) showed that women highly involved in sports reported a lower level of dissatisfaction with their body image compared to women with low involvement. Sports involvement refers to a love for sports and is the degree to which sport is important to a person (Gwinner and Swanson, 2003). Therefore, Involvement in sports is another variable that could be included in future research of body image dissatisfaction.

All materials, including the Somatomorphic Matrices, were provided in paper-and-pencil form. Application of the electronic form of the matrices would facilitate their ease of filling out and scoring (e.g., reducing the number of cases in which the visibility of the marked part of the image is weak, or making it impossible to mark two or more points on a single image which would invalidate determining the specific point on the scale that the participant felt best described his or her ideal or actual body).

## Conclusion

The results of the present research point to sex differences in dissatisfaction with some aspects of physique among students of the Faculty of Kinesiology. Specifically, differences in muscularity dissatisfaction were found, with men reporting higher dissatisfaction. No sex differences were found in body fat dissatisfaction. The result of hierarchical regression analyses showed that sex was the only statistically significant predictor of muscularity dissatisfaction out of all included variables. For body fat dissatisfaction, positive perfectionism was shown to be a negative predictor, and disordered eating attitudes were a positive predictor. The findings linking positive perfectionism and disordered eating attitudes with body image dissatisfaction (more precisely, body fat dissatisfaction) are in line with previous research such as Prnjak et al. (2019) or de Souza Fortes et al. (2015), and sex differences concerning muscularity dissatisfaction are in line with conventional findings (Grogan, 2016). This research contributes to understanding predictors of body image dissatisfaction in a specific population, such as the students of the Faculty of Kinesiology. Dissatisfaction with one’s body image could harm an individual’s well-being, justifying further research and exploring potential preventative measures.

## Data availability statement

The raw data supporting the conclusions of this article will be made available by the authors, without undue reservation.

## Ethics statement

The studies involving humans were approved by Ethics Committee of the Faculty of Kinesiology, University of Zagreb. The studies were conducted in accordance with the local legislation and institutional requirements. The ethics committee/institutional review board waived the requirement of written informed consent for participation from the participants or the participants’ legal guardians/next of kin because In the initial instructions, it was stated that participation in the research is voluntary and anonymous and that the data will be analyzed at the level of the entire group of respondents and will be used exclusively for research purposes. It was stated that the participants have the right to withdraw from the research at any time.

## Author contributions

VJ: Writing – original draft. MK: Writing – original draft. RB: Writing – original draft.

## Footnotes

^1^In the rest of this text, the term positive perfectionism will be used for the constructs that authors in the original studies labeled as perfectionistic strivings and adaptive perfectionism, while the term negative perfectionism will be used for the constructs authors labeled as perfectionist concerns and maladaptive perfectionism.

^2^In the rest of the study this variable will be referred to as disordered eating attitudes.

^3^Some participants compete at multiple levels (e.g., national competitions and international competitions for clubs). For such participants, the highest selected competition level was recorded. The gradation followed the order in which the levels are listed in the text.

^4^Regression models are robust even when the assumption of normality of distribution is violated to a lesser degree (see indices of skewness and kurtosis, Table 1; Ernst and Albers, 2017).

## References

[ref1] Ambrosi-Randić N. Pokrajac-Bulian A. (2005). Psychometric properties of the eating attitudes test and children’s eating attitudes test in Croatia. Eat Weight Disord. 10, e76–e82. doi: 10.1007/BF03327495, PMID: 16682865

[ref2] Anton S. D. Perri M. G. Riley J. R. (2000). Discrepancy between actual and ideal body images: impact on eating and exercise behaviors. Eat. Behav. 1, 153–160. doi: 10.1016/S1471-0153(00)00015-5 15001058

[ref3] Ben Ayed H. Yaich S. Ben Jemaa M. Ben Hmida M. Trigui M. Jedidi J.. (2021). What are the correlates of body image distortion and dissatisfaction among school-adolescents? Int. J. Adolesc. Med. Health 33:20180279. doi: 10.1515/ijamh-2018-0279, PMID: 31100056

[ref4] Besson H. Ekelund U. Luan J. May A. M. Sharp S. Travier N.. (2009). A cross-sectional analysis of physical activity and obesity indicators in European participants of the EPIC-PANACEA study. Int. J. Obes. 33, 497–506. doi: 10.1038/ijo.2009.25, PMID: 19223851

[ref5] Bradbury K. E. Guo W. Cairns B. J. Armstrong M. E. Key T. J. (2017). Association between physical activity and body fat percentage, with adjustment for BMI: a large cross-sectional analysis of UK biobank. BMJ Open 7:e011843. doi: 10.1136/bmjopen-2016-011843, PMID: 28341684 PMC5372047

[ref6] Cadegiani F. (2020). Overtraining syndrome in athletes: a comprehensive review and novel perspectives. Springer. Cham

[ref9001] Carmona J. Tornero-Quinones I. Sierra-Robles Á. (2015). Body image avoidance behaviors in adolescence: A multilevel analysis of contextual effects associated with the physical education class. Psychol Sport Exerc, 16, 70–78. doi: 10.1016/j.psychsport.2014.09.010

[ref7] Cash T. F. (1994). Body-image attitudes: evaluation, investment, and affect. Percept. Mot. Skills 78, 1168–1170. doi: 10.2466/pms.1994.78.3c.1168 7936939

[ref8] Cash T. F. (2004). Body image: past, present, and future. Body Image 1, 1–5. doi: 10.1016/s1740-1445(03)00011-1 18089136

[ref9] Cash T. F. Pruzinsky T. E. (1990). Body images: development, deviance, and change. Guilford press. New York

[ref10] Costa S. Coppolino P. Oliva P. (2016). Exercise dependence and maladaptive perfectionism: the mediating role of basic psychological needs. Int. J. Ment. Heal. Addict. 14, 241–256. doi: 10.1007/s11469-015-9586-6

[ref9002] Cox A. E. Ullrich-French S. Howe H. S. Cole A. N. (2017). A pilot yoga physical education curriculum to promote positive body image. Body image, 23, 1–8. doi: 10.1016/j.bodyim.2017.07.007 28818786

[ref11] Crocker J. Luhtanen R. K. Cooper M. L. Bouvrette A. (2003). Contingencies of self-worth in college students: theory and measurement. J. Pers. Soc. Psychol. 85, 894–908. doi: 10.1037/0022-3514.85.5.894, PMID: 14599252

[ref12] de Souza Fortes L. Ferreira M. E. de Oliveira S. M. Cyrino E. S. Almeida S. S. (2015). A socio-sports model of disordered eating among Brazilian male athletes. Appetite 92, 29–35. doi: 10.1016/j.appet.2015.05.005, PMID: 25963103

[ref13] de Souza Fortes L. Neves C. M. Filgueiras J. F. Almeida S. S. Ferreira M. E. C. (2013). Body dissatisfaction, psychological commitment to exercise and eating behavior in young athletes from aesthetic sports. Rev. Bras. Cineantropometria Desempenho Hum. 15, 695–704. doi: 10.5007/1980-0037.2013v15n6p695

[ref14] Demarest J. Allen R. (2000). Body image: gender, ethnic, and age differences. J. Soc. Psychol. 140, 465–472. doi: 10.1080/00224540009600485 10981375

[ref15] Enns M. W. Cox B. J. Clara I. (2002). Adaptive and maladaptive perfectionism: developmental origins and association with depression proneness. Personal. Individ. Differ. 33, 921–935. doi: 10.1016/S0191-8869(01)00202-1

[ref16] Ernst A. F. Albers C. J. (2017). Regression assumptions in clinical psychology research practice—a systematic review of common misconceptions. PeerJ 5:e3323. doi: 10.7717/peerj.3323, PMID: 28533971 PMC5436580

[ref17] Fairburn C. G. Cooper Z. Shafran R. (2003). Cognitive behaviour therapy for eating disorders: a “transdiagnostic” theory and treatment. Behav. Res. Ther. 41, 509–528. doi: 10.1016/S0005-7967(02)00088-8, PMID: 12711261

[ref18] Fang T. Liu F. (2022). A review on perfectionism. Open J. Soc. Sci. 10, 355–364. doi: 10.4236/jss.2022.101027

[ref19] Flett G. L. Hewitt P. L. (2002). “Perfectionism and maladjustment: an overview of theoretical, definitional, and treatment issues” in Perfectionism: Theory, research, and treatment. eds. Flett G. L. Hewitt P. L. (Washington: American Psychological Association), 5–31.

[ref20] Frost R. O. Marten P. Lahart C. Rosenblate R. (1990). The dimensions of perfectionism. Cogn. Ther. Res. 14, 449–468. doi: 10.1007/BF01172967

[ref21] Gao X. Zhong J. Li H. Pei Y. Li X. Zhang S.. (2023). The relationship between perfectionism, self-perception of orofacial appearance, and mental health in college students. Front. Public Health 11:1154413. doi: 10.3389/fpubh.2023.1154413, PMID: 37213631 PMC10196033

[ref22] Gardner R. M. Brown D. L. (2010). Body image assessment: a review of figural drawing scales. Personal. Individ. Differ. 48, 107–111. doi: 10.1016/j.paid.2009.08.017

[ref23] Garner D. M. Garfinkel P. E. (1979). The eating attitudes test: an index of the symptoms of anorexia nervosa. Psychol. Med. 9, 273–279. doi: 10.1017/S0033291700030762 472072

[ref24] Garner D. M. Olmsted M. P. Bohr Y. Garfinkel P. E. (1982). The eating attitudes test: psychometric features and clinical correlates. Psychol. Med. 12, 871–878. doi: 10.1017/S0033291700049163, PMID: 6961471

[ref25] Grammas D. L. Schwartz J. P. (2009). Internalization of messages from society and perfectionism as predictors of male body image. Body Image 6, 31–36. doi: 10.1016/j.bodyim.2008.10.002, PMID: 19046933

[ref26] Grogan S. (2006). Body image and health: contemporary perspectives. J. Health Psychol. 11, 523–530. doi: 10.1177/1359105306065013 16769732

[ref27] Grogan S. (2016). Body image: Understanding body dissatisfaction in men, women and children. Routledge. London

[ref28] Gwinner K. Swanson S. R. (2003). A model of fan identification: antecedents and sponsorship outcomes. J. Serv. Mark. 17, 275–294. doi: 10.1108/08876040310474828

[ref29] Hausenblas H. A. Downs D. S. (2001). Comparison of body image between athletes and nonathletes: a meta-analytic review. J. Appl. Sport Psychol. 13, 323–339. doi: 10.1080/104132001753144437

[ref30] Juarascio A. Manasse S. Clark K. E. Schaumberg K. Kerrigan S. Goldstein S. P.. (2020). Understanding the overlap and differences in terms describing patterns of maladaptive avoidance and intolerance of negative emotional states. Pers. Individ. Differ. 158:109859. doi: 10.1016/j.paid.2020.109859

[ref31] Kapetanović A. (2008). Self-esteem and perfectionism of female high school and university students. Available at: http://darhiv.ffzg.unizg.hr/id/eprint/626

[ref32] Kearney J. R. (2023). Conceptualizing adolescent social media usage through social comparison and self-discrepancy theories. Regent University. London

[ref33] Kesavachandran C. Bihari V. Mathur N. (2009). Can physical activity maintain normal grades of body mass index and body fat percentage? Int. J. Yoga 2, 26–29. doi: 10.4103/0973-6131.53839, PMID: 21234212 PMC3017964

[ref34] Kirkpatrick C. E. Lee S. (2023). Effects of Instagram body portrayals on attention, state body dissatisfaction, and appearance management behavioral intention. Health Commun. 38, 1430–1441. doi: 10.1080/10410236.2021.2010902, PMID: 34881654

[ref35] Kong P. Harris L. M. (2015). The sporting body: body image and eating disorder symptomatology among female athletes from leanness focused and nonleanness focused sports. J. Psychol. 149, 141–160. doi: 10.1080/00223980.2013.846291 25511202

[ref36] Kuterovac P. (2022). Psychological factor related to doping in sport. University of Zagreb. Zagreb

[ref37] Lacković-Grgin K. (1994). Samopoimanje mladih. Naklada Slap, Jastrebarsko.

[ref38] Lauri Korajlija A. (2004). Relationship between perfectionism and negative attributional style with depression and anxiety. Zagreb, University of Zagreb.

[ref39] Leng H. K. Phua Y. X. P. Yang Y. (2020). Body image, physical activity and sport involvement: a study on gender differences. Phys. Cult. Sport, Stud. Res. 85, 40–49. doi: 10.2478/pcssr-2020-0005

[ref40] Madigan D. J. Hill A. P. Anstiss P. A. Mallinson-Howard S. H. Kumar S. (2018b). Perfectionism and training distress in junior athletes: the mediating role of coping tendencies. Eur. J. Sport Sci. 18, 713–721. doi: 10.1080/17461391.2018.1457082, PMID: 29614917

[ref41] Madigan D. J. Stoeber J. Culley T. Passfield L. Hill A. P. (2018a). Perfectionism and training performance: the mediating role of other-approach goals. Eur. J. Sport Sci. 18, 1271–1279. doi: 10.1080/17461391.2018.1508503, PMID: 30102870

[ref42] Mayo C. George V. (2014). Eating disorder risk and body dissatisfaction based on muscularity and body fat in male university students. J. Am. Coll. Heal. 62, 407–415. doi: 10.1080/07448481.2014.917649, PMID: 24786836

[ref43] Meneguzzo P. Collantoni E. Meregalli V. Favaro A. Tenconi E. (2022). Addressing weight bias in the cisgender population: differences between sexual orientations. Nutrients 14:1735. doi: 10.3390/nu14091735, PMID: 35565703 PMC9099522

[ref44] Miles J. (2014). Tolerance and variance inflation factor. Hoboken: Wiley.

[ref45] Mutale G. J. Dunn A. K. Stiller J. Larkin R. (2016). Development of a body dissatisfaction scale assessment tool. New Sch. Psychol. Bull. 13, 47–57. Available at: https://irep.ntu.ac.uk/id/eprint/28326

[ref46] Muth J. L. Cash T. F. (1997). Body-image attitudes: what difference does gender make? J. Appl. Soc. Psychol. 27, 1438–1452. doi: 10.1111/j.1559-1816.1997.tb01607.x

[ref47] Novella J. Gosselin J. T. Danowski D. (2015). One size doesn’t fit all: new continua of figure drawings and their relation to ideal body image. J. Am. Coll. Heal. 63, 353–360. doi: 10.1080/07448481.2015.1040410, PMID: 25942358

[ref48] Nunnally J. C. (1978). Psychometric theory (2nd ed.). McGraw-Hill. New York

[ref49] O’Dea J. A. (2012). “Body image and self-esteem” in Encyclopedia of body image and human appearance. ed. Cash T. F. (Amsterdam: Elsevier Academic Press), 141–147.

[ref50] Ouyang Y. Wang K. Zhang T. Peng L. Song G. Luo J. (2020). The influence of sports participation on body image, self-efficacy, and self-esteem in college students. Front. Psychol. 10:3039. doi: 10.3389/fpsyg.2019.03039, PMID: 32116869 PMC7012809

[ref51] Petrie T. A. Greenleaf C. A. (2007). “Eating disorders in sport: from theory to research to intervention” in Handbook of sport psychology. eds. Tenenbaum G. Eklund R. C. (Hoboken: John Wiley & Sons, Inc), 352–378.

[ref52] Pokrajac-Bulian A. Stubbs L. Ambrosi-Randić N. (2004). Different aspects of body image and eating habits in adolescence. Psychol. Top. 13, 91–104. Available at: https://hrcak.srce.hr/12656

[ref53] Pokrajac-Bulian A. Živčić-Bećirević I. Vukmanović S. Forbes G. (2005). Body dissatisfaction and eating habits in college students and their mothers. Psychol. Top. 14, 57–70. Available at: https://hrcak.srce.hr/4829

[ref54] Prnjak K. Jukic I. Tufano J. J. (2019). Perfectionism, body satisfaction and dieting in athletes: the role of gender and sport type. Sports 7:181. doi: 10.3390/sports7080181, PMID: 31344910 PMC6723820

[ref55] R Core Team (2022). R: a language and environment for statistical computing. R Foundation for Statistical Computing, Vienna, Austria

[ref56] Rodin J. Silberstein L. Striegel-Moore R. (1984). “Women and weight: a normative discontent” in Nebraska symposium on motivation. ed. Sonderegger T. B., Psychology and gender (Nebraska: University of Nebraska Press), 267–307. 6398857

[ref57] Rosenberg M. (1965). Society and the adolescent self-image. Princeton, NJ: Princeton University Press.

[ref58] Rosenberg M. Schooler C. Schoenbach C. Rosenberg F. (1995). Global self-esteem and specific self-esteem: different concepts, different outcomes. Am. Sociol. Rev. 60, 141–156. doi: 10.2307/2096350

[ref59] Sherry S. B. Vriend J. L. Hewitt P. L. Sherry D. L. Flett G. L. Wardrop A. A. (2009). Perfectionism dimensions, appearance schemas, and body image disturbance in community members and university students. Body Image 6, 83–89. doi: 10.1016/j.bodyim.2008.12.002, PMID: 19200791

[ref60] Smith A. R. Hawkeswood S. E. Bodell L. P. Joiner T. E. (2011). Muscularity versus leanness: an examination of body ideals and predictors of disordered eating in heterosexual and gay college students. Body Image 8, 232–236. doi: 10.1016/j.bodyim.2011.03.005, PMID: 21561818 PMC3124584

[ref61] Soares Filho L. C. Batista R. F. L. Cardoso V. C. Simões V. M. F. Santos A. M. Coelho S. J. D. D. A. C.. (2020). Body image dissatisfaction and symptoms of depression disorder in adolescents. Braz. J. Med. Biol. Res. 54:e10397. doi: 10.1590/1414-431X202010397 33295537 PMC7727113

[ref62] Stewart S. J. F. (2021). The role of sociocultural factors on body weight, weight bias and health behaviours. Guildford: University of Surrey.

[ref63] Stoeber J. (2011). The dual nature of perfectionism in sports: relationships with emotion, motivation, and performance. Int. Rev. Sport Exerc. Psychol. 4, 128–145. doi: 10.1080/1750984X.2011.604789

[ref64] Stoeber J. Otto K. Pescheck E. Becker C. Stoll O. (2007). Perfectionism and competitive anxiety in athletes: differentiating striving for perfection and negative reactions to imperfection. Personal. Individ. Differ. 42, 959–969. doi: 10.1016/j.paid.2006.09.006

[ref65] Sundgot-Borgen C. Sundgot-Borgen J. Bratland-Sanda S. Kolle E. Torstveit M. K. Svantorp-Tveiten K. M.. (2021). Body appreciation and body appearance pressure in Norwegian university students comparing exercise science students and other students. BMC Public Health 21, 532–511. doi: 10.1186/s12889-021-10550-0, PMID: 33740918 PMC7977603

[ref66] Talbot D. Cass J. Smith E. (2020). Male figural rating scales: a critical review of the literature. Behav. Chang. 37, 59–73. doi: 10.1017/bec.2020.5

[ref67] Talbot D. Mahlberg J. Cunningham M. L. Pinkus R. T. Szabo M. (2023). The Somatomorphic matrix-female: more evidence for the validity of bidimensional figural rating scales for women. J. Clin. Psychol. 79, 477–496. doi: 10.1002/jclp.23420 36000930

[ref68] Talbot D. Smith E. Cass J. Griffiths S. (2019). Development and validation of the new Somatomorphic matrix–male: a figural rating scale for measuring male actual–ideal body discrepancy. Psychol. Men Masc. 20, 356–367. doi: 10.1037/men0000165

[ref69] Teixeira M. D. Pereira A. T. Marques M. V. Saraiva J. M. Macedo A. F. D. (2016). Eating behaviors, body image, perfectionism, and self-esteem in a sample of Portuguese girls. Braz. J. Psychiatry 38, 135–140. doi: 10.1590/1516-4446-2015-1723, PMID: 26870911 PMC7111361

[ref70] Terry-Short L. A. Owens R. G. Slade P. D. Dewey M. E. (1995). Positive and negative perfectionism. Personal. Individ. Differ. 18, 663–668. doi: 10.1016/0191-8869(94)00192-U

[ref71] Tiggemann M. (2005). Body dissatisfaction and adolescent self-esteem: prospective findings. Body Image 2, 129–135. doi: 10.1016/j.bodyim.2005.03.006 18089181

[ref72] Tylka T. L. (2004). The relation between body dissatisfaction and eating disorder symptomatology: an analysis of moderating variables. J. Couns. Psychol. 51, 178–191. doi: 10.1037/0022-0167.51.2.178

[ref73] Tylka T. L. (2011). Refinement of the tripartite influence model for men: dual body image pathways to body change behaviors. Body Image 8, 199–207. doi: 10.1016/j.bodyim.2011.04.008, PMID: 21664886

[ref74] Valls M. Bonvin P. Chabrol H. (2013). Association between muscularity dissatisfaction and body dissatisfaction among normal-weight French men. J. Men’s Health 10, 139–145. doi: 10.1089/jomh.2013.0005

[ref75] Van Den Berg P. A. Mond J. Eisenberg M. Ackard D. Neumark-Sztainer D. (2010). The link between body dissatisfaction and self-esteem in adolescents: similarities across gender, age, weight status, race/ethnicity, and socioeconomic status. J. Adolesc. Health 47, 290–296. doi: 10.1016/j.jadohealth.2010.02.004, PMID: 20708569 PMC2923488

[ref76] Vannucci A. Ohannessian C. M. (2018). Body image dissatisfaction and anxiety trajectories during adolescence. J. Clin. Child Adolesc. Psychol. 47, 785–795. doi: 10.1080/15374416.2017.1390755, PMID: 29087230 PMC6072626

[ref77] Wade T. D. Tiggemann M. (2013). The role of perfectionism in body dissatisfaction. J. Eat. Disord. 1, 1–6. doi: 10.1186/2050-2974-1-2, PMID: 24764525 PMC3776202

[ref78] Welch E. Miller J. L. Ghaderi A. Vaillancourt T. (2009). Does perfectionism mediate or moderate the relation between body dissatisfaction and disordered eating attitudes and behaviors? Eat. Behav. 10, 168–175. doi: 10.1016/j.eatbeh.2009.05.002 19665100

[ref79] Yang F. Qi L. Liu S. Hu W. Cao Q. Liu Y.. (2022). Body dissatisfaction and disordered eating behaviors: the mediation role of smartphone addiction and depression. Nutrients 14:1281. doi: 10.3390/nu14061281, PMID: 35334936 PMC8955505

[ref80] Zogmaister C. Maricutoiu L. P. (2022). Mirror, mirror on the wall, tell me that I have succeeded at it all: self-esteem and the defensive mechanisms against failure. Soc. Psychol. Educ. 25, 1221–1248. doi: 10.1007/s11218-022-09723-5

